# Mechanisms of SOSS‐Integrator–PP2A complex in attenuating R‐loops and promoting genome stability

**DOI:** 10.1002/ctm2.1519

**Published:** 2024-01-16

**Authors:** Jingwen Wang, Conglin Xu, Zhen Zhang, Fei Xavier Chen

**Affiliations:** ^1^ Department of Radiation Oncology Shanghai Medical College Fudan University Shanghai Cancer Center, Fudan University Shanghai China; ^2^ Department of Oncology, Shanghai Medical College Fudan University Shanghai Cancer Center, Fudan University Shanghai China; ^3^ Shanghai Key Laboratory of Anesthesiology and Brain Functional Modulation, Clinical Research Center for Anesthesiology and Perioperative Medicine, Translational Research Institute of Brain and Brain‐Like Intelligence, Shanghai Fourth People's Hospital, School of Life Sciences and Technology Tongji University Shanghai China; ^4^ Institutes of Biomedical Sciences Fudan University Shanghai China

1

In metazoans, transcription is a finely regulated dynamic process, involving initiation, elongation and termination. Following transcription initiation, RNA polymerase II (Pol II) is halted at the proximal promoter region, with the potential for productive elongation or premature termination signaled by further events.^1^ Recent studies have revealed a previously undocumented role for premature termination as a gene regulatory strategy, largely facilitated by the Integrator–PP2A (INTAC) complex.^2^, ^3^, ^4^ The complex fulfills its role through the dephosphorylation of the carboxy‐terminal domain of Pol II and elongation factors such as SPT5, and plays a significant role in both disease and development. Nevertheless, the mechanisms governing the regulation of this complex for premature termination and its potential interactions with other chromatin‐based events remain unclear.

R‐loops, comprising RNA–DNA hybrids and a displaced strand of DNA, formed during transcription are frequently observed at active promoters owing to a high occupancy of paused Pol II.^5^ Deregulation of R‐loops has been associated with the formation of DNA double‐strand breaks (DSBs), transcription elongation defects and genome instability, accentuating the need for appropriate regulation of R‐loops. Nonetheless, the mechanisms by which the transcription machinery governs R‐loops and safeguards genome stability remain incompletely elucidated.

In the recent study,^6^ we identified a stable association between the genome‐stability regulator sensor of single‐stranded DNA (SOSS) and the INTAC complex by mass spectrometry and co‐immunoprecipitation. Through SSB1‐mediated recognition of single‐stranded DNA, the SOSS–INTAC was recruited to actively transcribe regions and stimulate promoter‐proximal termination of transcription. Furthermore, the researchers demonstrated that SSB1‐regulated R‐loops at promoters modulate SOSS–INTAC recruitment to chromatin, which in turn restricts R‐loop accumulation and thereby ensures genome stability. To understand better how SSB1 determines SOSS–INTAC localization, the team conducted immunofluorescence and observed the formation of nuclear puncta. It was discovered that SSB1 can form a liquid‐like SOSS–INTAC condensation via its intrinsically disordered region, which reduces cellular R‐loop levels and maintains genome integrity. Cancer‐derived mutations in SSB1 disrupt the formation of SOSS–INTAC condensation and impair its function of controlling R‐loops and genome stability, indicating its possible involvement in oncogenesis.

R‐loops have been considered transcriptional by‐products, which are detrimental to cellular physiology and threaten genomic stability if not removed properly. Beyond these connections, R‐loops are now mechanistically related to innate immune response, as reported by Crossley et al.^7^ The researchers discovered a stable structure of R‐loops in the cytoplasm that is similar to the formation found in nuclei. The cytoplasmic hybrids can bind to the pattern recognition receptors cGAS and TLR3, activating IRF3 and thereby inducing cell apoptosis. The findings support the view that the RNA–DNA hybrids are immunogenic species, and connect the accumulation of R‐loops with cell death via innate immune response. Consequently, this study suggests that activation of the innate immune pathway could indicate a distinct pathological role of R‐loops differed from the typical DNA damage response.

The breakthrough discovery that the SOSS–INTAC complex regulates promoter‐proximal termination of transcription and maintains genome stability by modulating R‐loops enhances our understanding of transcriptional regulation. Although there is a long way to go before a treatment targeting the SOSS–INTAC complex or R‐loops, our study provides valuable insights into clinical application. First, the SOS–INTAC complex and R‐loops have the potential to serve as biomarkers of genome instability induced by replication or transcription stress. Methods currently used to assess the genome‐wide distribution of R‐loops rely on affinity purification, a process that requires large inputs and is challenging to perform, thus restricting its application to clinical samples. It is imperative to develop simplified assays to quantify R‐loop accumulation in patients. This will aid in identifying more R‐loop abnormalities related to diseases.

Secondly, associating the SOSS–INTAC complex and R‐loops with clinical treatment is crucial. Deregulation of R‐loops significantly contributes to genome instability, which can be targeted therapeutically with DNA damage response (DDR) pathway inhibitors.^8^, ^9^ Thus, investigating the correlation between R‐loop levels and the response to DDR pathway inhibitors would be worthwhile. Patients with mutations in the breast cancer gene generally exhibit sensitivity to PARP inhibitors. As such, it is important to explore the regulatory function of R‐loops specifically in this context, where DNA damage repair is almost completely impaired. In addition, DNA mismatch repair deficiency (dMMR) serves as a biomarker for immune checkpoint inhibitors,^10^ but certain MMR‐proficient (pMMR) tumors also show responsiveness to immunotherapy. The correlation between immunotherapy sensitivity in pMMR patients and R‐loop accumulation induced by transcription or replication stress needs further exploration.

Moreover, targeting R‐loops is a potentially effective method to achieve precise transcriptional regulation. Since the technology to transcriptionally regulate the expression of specific genes is not yet established, it is worth exploring the targeting of the R‐loop generated by genes of interest with the guidance of the SOSS–INTAC complex. This has the potential to close the gap in accurate transcriptional regulation and holds significant promise for clinical applications. Figure 1.

**FIGURE 1:**
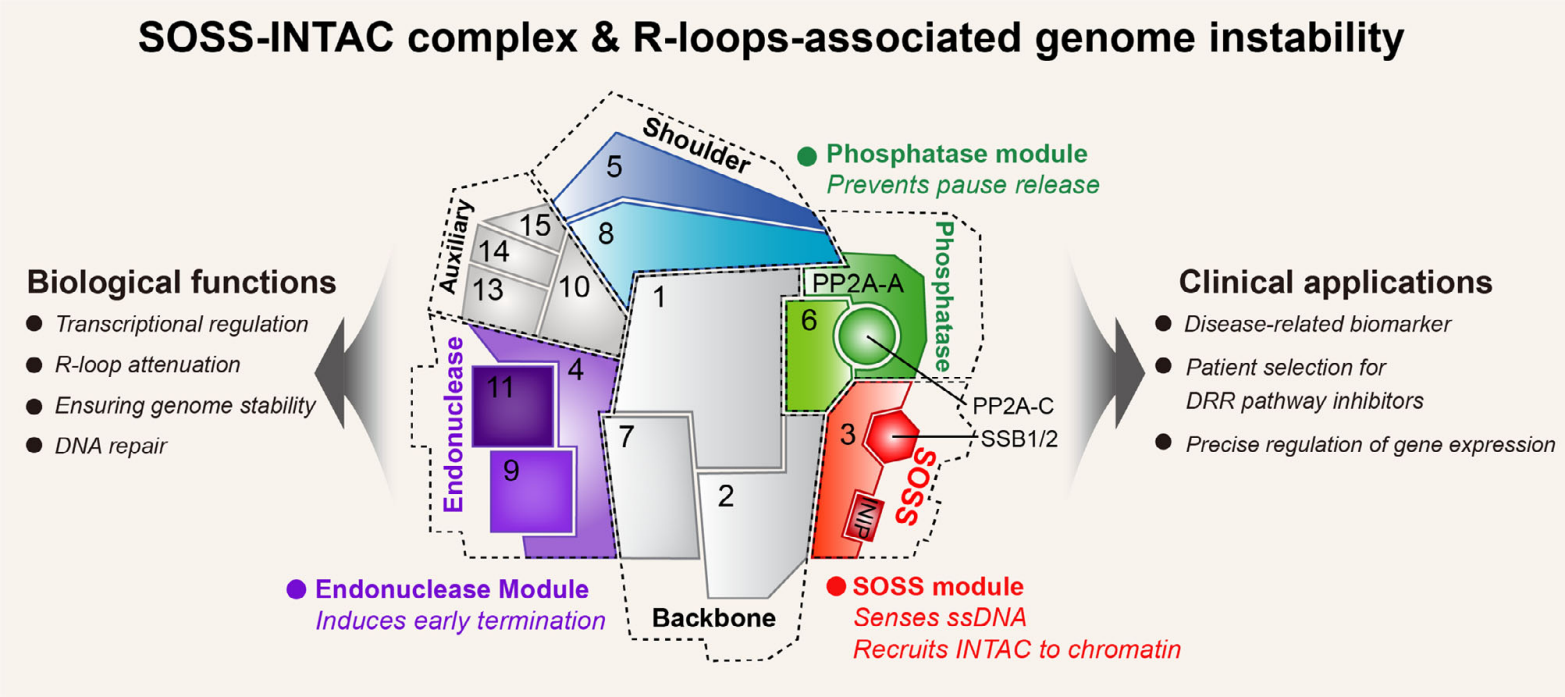
Schematic illustration of the biological functions and clinical applications of the single‐stranded DNA‐Integrator–PP2A (SOSS–INTAC) complex. The SOSS–INTAC complex, pivotal in genome stability, orchestrates transcriptional regulation by modulating R‐loops. Key modules of the complex include the endonuclease module, responsible for inducing early termination of DNA damage, and the phosphatase module, which inhibits the release of the replication pause, and therefore plays a role in transcriptional regulation and R‐loop attenuation. The SOSS module recognizes single‐stranded DNA (ssDNA) and recruits INTAC to chromatin, which is critical for DNA repair and maintenance of genome stability. Potential clinical implications highlight the complex's potential as a disease‐related biomarker of genomic instability, its role in patients' selection for DNA repair response (DRR) pathway inhibitors, and its importance in the precise regulation of gene expression. These applications hold promise for disease diagnosis and therapeutic targeting, particularly in tailoring treatments to R‐loop‐related disorders, uncovering their association with drug response, and exploring avenues for precise transcriptional regulation.

In summary, our recent study on R‐loops contributes to the understanding of regulatory R‐loops as modulators of gene expression and genomic stress. The study successfully bridges the gap between transcriptional regulation and genome stability. Going forward, it will be critical to examine how the SOSS–INTAC complex and R‐loops function in genome stability in specific cellular, tissue and disease contexts. This exploration will be fundamental for the development of new strategies aimed at combating R‐loop‐associated defects.

## AUTHOR CONTRIBUTIONS

**Jingwen Wang**: Conceptualization; Methodology; Investigation; Writing – Original Draft Preparation; Writing – Review & Editing; Visualization. **Conglin Xu**: Conceptualization; Writing – Review & Editing. **Zhen Zhang**: Conceptualization; Writing – Review & Editing. **Fei Xavier Chen**: Conceptualization; Writing – Review & Editing; Visualization; Supervision; Supervision.

## CONFLICT OF INTEREST STATEMENT

The authors declare no conflict of interest.

## ETHICS STATEMENT

Not Applicable.
